# The prevalence and characteristics of non-functioning and autonomous cortisol secreting adrenal incidentaloma after patients’ stratification by body mass index and age

**DOI:** 10.1186/s12902-020-00599-0

**Published:** 2020-07-31

**Authors:** Ana Podbregar, Andrej Janez, Katja Goricar, Mojca Jensterle

**Affiliations:** 1 Department of Internal Medicine, Faculty of Medicine, University of Ljubljana, Ljubljana, Slovenia; 2 University Rehabilitation Institute Republic of Slovenia, Ljubljana, Slovenia; 3 Department of Endocrinology, Diabetes and Metabolic Disease, University Medical Center Ljubljana, Ljubljana, Slovenia; 4 Pharmacogenetics Laboratory, Institute of Biochemistry, Faculty of Medicine, University of Ljubljana, Ljubljana, Slovenia

**Keywords:** Adrenal incidentaloma, Obesity, Autonomous cortisol secreting adrenal incidentaloma, Non-functioning adrenal incidentaloma

## Abstract

**Background:**

The escalating prevalence of adrenal incidentaloma (AI) has been associated with the improvement of radiologic techniques and widespread imaging in aging population. It is currently unclear whether patients with obesity more likely develop AI and the current rise in the prevalence of AI could be at least partly associated with the respective rise in obesity. We compared the prevalence and characteristics of non-functional (NF) and autonomous cortisol secreting (ACS) adrenal incidentalomas (AIs) after the study population was stratified by different body mass indexes (BMI) and age groups.

**Methods:**

Retrospective cross-sectional study comprising of 432 patients (40.6% male, 59.4% female) with NFAI (*N* = 290) and ACS (*N* = 142), of median age 63.4 (54.0–71.6) years and median BMI 28.6 (25.5–31.7) kg/m^2^. The data collection contained 11.132 points including demographic, anthropometric, radiologic, hormonal and metabolic parameters.

**Results:**

We observed 68–87% higher prevalence of AI across different age groups in NFAI and ACS in obese/overweight compared to normal weight subjects. Patients with ACS were older (*P* = 0.008), with higher basal cortisol (*P* < 0.001), lower basal DHEAS (*P* = 0.001), lower suppression DHEAS (*P* = 0.027) and higher aldosterone (*P* = 0.039). AIs with ACS were larger than NFAI (*P* < 0.001). Interestingly, ACS group had lower body mass (*P* = 0.023) and did not differ in BMI, blood pressure, heart rate, lipid profile, fasting glucose and presence of diabetes mellitus type 2 when compared to NFAI., By contrast to the similarity of metabolic profiles in ACS and NFAI, some components of adverse metabolic traits were rather associated with higher BMI and older age, in particular in NFAI.

**Conclusion:**

The prevalence of NFAI and ACS were significantly higher in overweight/obese subgroup across the age distribution. Stratification by age and BMI displayed significant differences in some metabolic traits, in particular in NFAI.

## Background

Adrenal incidentaloma (AI) is an asymptomatic adrenal mass detected on imaging not performed for suspected adrenal disease [1]. It has recently become increasingly common finding reported in 3–5% in general population [2–4] and up to 10% in elderly [5, 6], as compared to mean prevalence of 0.5 to 2% in studies from 80s and 90s [7]. This escalating prevalence has been associated with the improvement of radiologic techniques and widespread imaging in aging population [8].

The majority of AIs are characterized as nonfunctional (NFAI) [1, 9, 10]. Only less than 10% secrete excess amounts of hormones, most frequently autonomous cortisol secretion (ACS) [10, 11]. Although patients with AI, by definition, do not show any signs and symptoms of adrenal hormonal excess [12], insulin resistance (IR) hypertension, dyslipidemia, glucose intolerance and obesity in ACS are clearly associated with the slight cortisol hypersecretion [11, 13, 14]. Moreover, the increased frequencies of IR, impaired glucose homeostasis and metabolic syndrome were reported also in patients with NFAI when compared to those with normal adrenal imaging [15]. It was speculated that the existence of minimal hormonal secretion not detectable by current diagnostic methods might led to adverse metabolic profile in NFAI [3]. On the contrary, IR itself has been linked with anabolic and mitogenic effects on adrenal cortex through the activation of insulin and insulin like growth factor 1 (IGF 1) receptors [3].

It is currently unclear whether patients with obesity and IR more likely develop AI and the current rise in the prevalence of AI could be at least partly associated with the respective rise in obesity.

## Methods

### Aim

We aimed to evaluate the prevalence and characteristics of NFAI and ACS after the study population stratification by different BMI and age groups.

### Design and setting of the study

We conducted a retrospective cross-sectional study including all patients with AI referred to University Medical Centre from January 2005 to January 2012. After that period, the majority of patients with AI were managed in our outpatients’ clinics. The study was conducted in accordance with the Declaration of Helsinki, approved by the National Ethical Committee.

### Study population

**Fig. 1:**
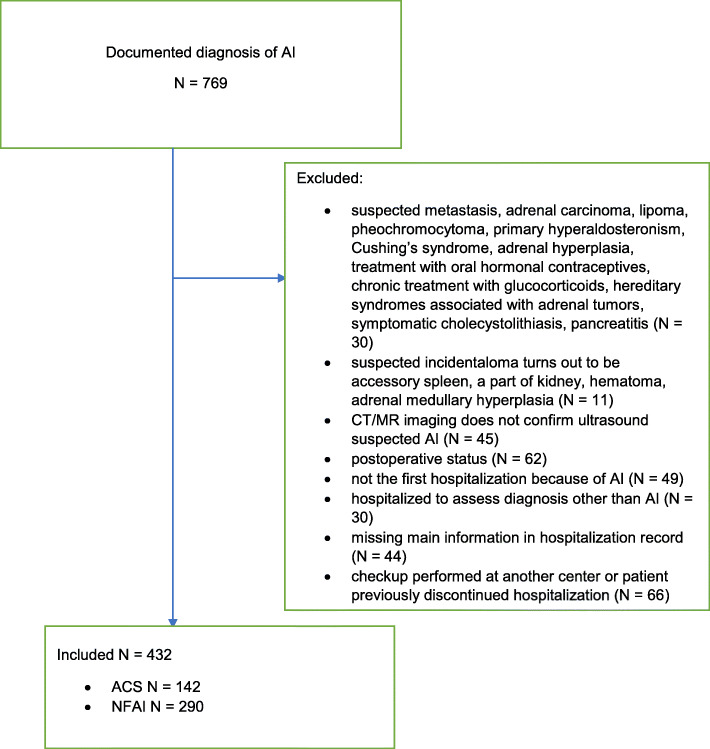
Flowchart of the study N - number of patients. AI – adrenal incidentaloma. CT - computed tomography. MR- magnetic resonance. NFAI - non-functioning adrenal incidentalomas. ACS - adrenal incidentalomas with autonomous cortisol secretion

We identified 769 adult patients with documented diagnosis of AI. The medical records of all patients were reviewed by the authors to confirm the diagnosis of AI based on European Society of Endocrinology Clinical Practice Guideline for management of AIs [1] and divided them into NFAI and ACS. NFAI was considered when cortisol after overnight dexamethasone (ODST) suppression test was < 50 nmol/l, ACS when cortisol was ≥50 nmol/l [1] and there were no typical clinical signs of Cushing’s syndrome. One to three consultants that were experienced in the diagnosis of Cushing syndrome excluded typical catabolic signs of hypercortisolism. Included AI had radiological features of lipid-rich, benign adrenal adenoma as defined with Hounsfield units (HU) of ≤10 for noncontrast CT [16] or a relative washout > 40% and an absolute washout > 60% for contrast-enhanced washout CT, when an attenuation value for unenhanced CT was > 10 HU [17] or benign characteristic in magnetic resonance imaging (MRI) [18]. We excluded suspected metastasis, adrenal carcinoma, lipoma, pheochromocytoma, adrenal hyperplasia and patients with primary hyperaldosteronism. In addition, we excluded all patients with symptomatic cholecystolithiasis and pancreatitis. Among exclusion criteria were also patients under treatment with the drugs that significantly interfere with measured variables including oral hormonal contraceptives, glucocorticoids, mineralocorticoid antagonists and potassium-wasting diuretics. Some less interfering antihypertensive medications and lipid lowering agents were not consistently discontinued before measuring aldosterone and PRA. Subjects with hereditary syndromes associated with adrenal tumors were also excluded. The Flowchart of the study is presented in Fig. 1.

### Data collection

The following data were collected from the hospital documentation system: patient gender, age at diagnosis, weight, height, method of radiological assessment of AI (computed tomography, magnetic resonance), size of AI, site of AI (left, right, or bilateral), Hounsfield units, blood pressure, heart rate, baseline cortisol and cortisol after ODST, baseline ACTH, baseline DHEAS, DHEAS after ODST, aldosterone, PRA, TSH, fasting glucose, lipids (cholesterol, HDL, LDL, triglycerides). Normal levels of baseline cortisol were defined as 138–690 nmol/l, cortisol after ODST < 50 nmol/l, ACTH < 10.2 pmol/l, DHEAS 3.6–12.9 μmol/L, aldosterone 0.134–0.751 nmol/l, PRA 0.6–4.84 μg/l/h, TSH 0.59–4.29 mE/l, fasting glucose 3.6–6.1 mmol/liter, cholesterol < 5.2 mmol/liter, HDL > 1 mmol/liter, LDL < 3.4 mmol/liter, triglycerides < 1.7 mmol/liter. Among additional biochemical parameters we included creatinine, natrium and leucocytes count since they have been included into the risk estimator for adrenal tumor functionality [19]. The data collection contained 11,132 points.

Cortisol was determined using IMMULITE® 2000 Cortisol assay. Intra-assay variations were ≤ 15% and inter-assay variations were ≤ 20% for the applied methods. DHEAS was measured by specific double antibody RIA using 125 I-labeled hormones (Diagnostic Systems Laboratories, Webster, TX, USA). Intra-assay coefficient variation (CV) for DHEAS ranged from 4.9 to 9.8% and inter-assay CV from 7.9 to 13.0%. Aldosterone was determined using ACTIVE® Aldosterone RIA for serum samples. Intra-assay variations were ≤ 4.5% and inter-assay variations were ≤ 9.8% for the applied methods. PRA was determined using Angiotensin I RIA KIT for serum samples. Intra-assay variations were ≤ 8% and inter-assay variations were ≤ 10% for the applied methods. ACTH was determined using IMMULITE® 2000 ACTH system. Intra-assay variations were ≤ 9.5% and inter-assay variations were ≤ 10% for the applied methods. TSH was determined using ADVIA Centaur CP TSH3-Ultra assay. Intra-assay variations were ≤ 16.7% and inter-assay variations were ≤ 22.3% for the applied methods. Fasting glucose levels were determined using the standard glucose oxidase method (Beckman Coulter Glucose Analyzer, Beckman Coulter Inc., CA, USA). Lipids were determined using Adiva 1800, Siemens analyzer. Intra-assay variations ranged from 1.6 to 6.3%, and inter-assay variations ranged from 5.8 to 9.6% for the applied methods. Creatinine, natrium, leucocytes count were assessed with routine biochemical evaluation after 12-h overnight fast. CT evaluation included maximum diameter of incidentaloma, laterality, presence of necrosis, calcifications, atypical characteristics. MR report included evaluation of maximum diameter of the tumor and laterality. BMI was calculated as the weight in kilograms divided by square of height in meters. Blood pressure was measured with sphygmomanometer using appropriate cuff size.

### Statistical analysis

The results for continuous variables are presented as means and standard deviation (SD) or median and interquartile range. The results for categorical variables are presented as frequencies. Spearman’s rho was used to assess correlation between continuous variables, while Fisher’s exact test was used to assess the association between categorical variables. To compare continuous variables between different groups, nonparametric Mann-Whitney test or Kruskal-Wallis and pairwise comparisons with post hoc Bonferroni corrections were used. To calculate prevalences, population data from SI-STAT (https://pxweb.stat.si/) database was used. *P* values below 0.05 were considered statistically significant. Statistical analysis was performed using IBM SPSS Statistics, version 21.0 (IBM Corporation, Armonk, New York, USA).

## Results

### Characteristics of study population

The study population consisted of 432 patients (179 (41.4%) male, 253 (58.6%) female) of median age 63.4 (54.0–71.6) years, median body mass 77.6 (67.4–88.8) kg and median BMI 28.6 (25.5–31.7) kg/m^2^. We identified 290 patients with NFAI and 142 with ACS, among which 128 had cortisol after overnight dexamethasone (ODST) suppression test between 50 nmol/l and 138 nmol/l and 14 had cortisol levels post dexamethasone > 138 nmol/L.

In majority of subjects, AI was diagnosed by CT (388 (92.2%)), in the rest by MRI [11 missing data]. 183 (43.9%) of patients presented with right-sided AI, 147 (35.3%) with left sided AI. In 87 (20.9%) AI was observed bilaterally [15 missing data]. Median size of right sided AI was 25 [19–30] mm and of left sided was 20 [15–30] mm. Size of the AI did not correlate with the presence of diabetes mellitus type 2 (presented in 52 (12.0%) patients), nor in NFAI or in ACS group (both *P*-values > 0.05).

### Comparison between unilateral and bilateral AIs

In NFAI group 243 subjects had one-side AI, 47 had bilateral adrenal mass. One-sided NFAI did not differ from bilateral NFAI in any of the anthropometric, clinical, hormonal, biochemical or radiologic characteristics (all *P*-values > 0.05). In ACS group 102 subject had one-side AI, 40 had bilateral adrenal mass. One-sided ACS had lower cholesterol (*P* = 0.021) and left-sided AI in patients with bilateral ACS were smaller than left-sided AI in patients with unilateral ACS (*P* = 0.025). One-sided ACS did not differ from bilateral ACS in any other anthropometric, clinical, hormonal, biochemical or radiologic characteristics (all *P*-values > 0.05).

### Comparison between NFAI and ACS

The comparison of selected parameters between NFAI and ACS is outlined in Table 1. Patients with ACS were older (*P* = 0.008), with higher basal cortisol level (*P* < 0.001), lower basal DHEAS (*P* = 0.001), lower DHEAS after ODST (*P* = 0.027) and higher aldosterone (*P* = 0.039). They had slightly lower weight (*P* = 0.023). AIs with ACS were significantly larger than NFAI (*P* < 0.001). There was no significant difference in gender distribution, ACTH, PRA, TSH, creatinine, sodium and leukocytes between patients with ACS and NFAI (all *P*-values > 0.05). We observed no significant difference between NFAI and ACS groups regarding BMI, blood pressure, heart rate, lipid profile, fasting glucose and presence of diabetes mellitus type 2 (all *P*-values > 0.05).

**Table 1:** Comparison of hormonal, antropometric, clinical, radiologic, biochemical and metabolic parameters between NFAI and ACS

Variables	NFAI (***N*** = 290)	ACS (***N*** = 142)	P
Age (yr)	61.76 ± 11.13 [62.67 (53.34–70.47)]	64.90 ± 12.08 [66.1 (54.94–74.8)]	**0.008**
Maximal tumor size (mm)	23.7 ± 11.1 [22.2 (16–29.0)]	30.3 ± 13.3 [30 (22.5–35.5)]	**< 0.001**
Size of rightsided tumor (mm)	23.7 ± 9.8 [23 (17.3–28)]	28.3 ± 11.1 [28.5 (20.0–35.0)]	**< 0.001**
Size of leftsided tumor (mm)	21.5 ± 11.9 [20 (14–25)]	28.4 ± 15.5 [26 (20–35)]	**< 0.001**
Basal serum cortisol (nmol/l)	398.33 ± 128.24 [384 (320–451.8)]	481.02 ± 240.00 [457 (350.25–556.25)]	**< 0.001**
Serum cortisol after ODST (nmol/l)^a^	32.8 ± 6.7 [29.4 (27.6–37.6)]	108.6 ± 277.4 [67.8 (55.9–87.4)]	**< 0.001**
ACTH (pmol/l)	2.22 ± 1.42 [1.90 (1.17–2.81)]	3.34 ± 7.54 [1.58 (1.11–2.58)]	0.398
Basal DHEAS (μmol/L)	1.92 ± 1.29 [1.7 (1–2.43)]	1.09 ± 0.94 [0.9 (0.4–1.4)]	**< 0.001**
DHEAS after ODST (μmol/L)	0.93 ± 0.76 [0.7 (0.4–1.2)]	0.74 ± 0.67 [0.5 (0.3–1)]	**0.027**
Aldosteron (nmol/l)	0.20 ± 0.13 [0.17 (0.11–0.25)]	0.26 ± 0.21 [0.21 (0.12–0.3)]	**0.039**
Plasma Renin Activity – PRA (μg/l/h)	1.25 ± 2.29 [0.38 (0.16–1.37)]	1.68 ± 3.19 [0.59 (0.19–1.63)]	0.132
TSH (mE/l)	2.93 ± 2.50 [1.68 (1.13–5.43)]	2.75 ± 2.57 [1.41 (0.6–5.73)]	0.186
Body mass (kg)	80.51 ± 15.92 [79.6 (68.75–89.45)]	77.15 ± 16.37 [74.8 (65.78–87.18)]	**0.023**
BMI (kg/m^2^)	29.33 ± 5.62 [28.59 (25.61–32.13)]	28.56 ± 5.37 [27.77 (25.48–31.28)]	0.287
Systolic blood pressure (mm Hg)	138.36 ± 18.94 [140 (124–151)]	141.63 ± 24.02 [140 (125–155)]	0.339
Diastolic blood pressure (mm Hg)	77.74 ± 11.51 [76 (70–85)]	77.72 ± 12.02 [75 (70–85)]	0.896
Fasting glucose (mmol/liter)	5.75 ± 1.40 [5.4 (4.9–6.13)]	5.78 ± 1.71 [5.4 (5–6.1)]	0.876
Total cholesterol (mmol/liter)	5.14 ± 1.14 [5.1 (4.4–5.8)]	5.09 ± 1.17 [5.1 (4.3–5.75)]	0.716
HDL (mmol/liter)	1.31 ± 0.36 [1.3 (1.1–1.5)]	1.32 ± 0.41 [1.2 (1–1.5)]	0.849
LDL (mmol/liter)	3.15 ± 0.98 [3.1 (2.4–3.78)]	3.05 ± 1.00 [3 (2.5–3.7)]	0.473
Triglycerides (mmol/liter)	1.75 ± 0.95 [1.5 (1.2–2.1)]	1.77 ± 0.88 [1.6 (1.2–2.1)]	0.622

^a^for 70 patients, data were provided as below 27.6, for 20 below 28 and for 1 below 31.3

Data are given as mean ± SD [Median (25–75%)]

## Stratification of patients with NFAI and ACS by age

### NFAI group stratified by age

In NFAI group, younger patients have lower blood pressure, lower body mass, LDL, fasting glucose, lower creatinine, basal DHEAS and DHEAS after ODST in comparison with older patients. There were significantly more younger women than man (*P* = 0.032). The data are provided in Table 2. TSH levels differed significantly among age groups (*P* < 0.001), with highest values in patients aged 60–69 years and lowest values in patients over 79 years. There were no differences in leukocyte concentration (*P* = 0.958).

**Table 2:** Stratification of hormonal, antropometric, clinical, radiologic, biochemical and metabolic parameters in NFAI patients by age

Variable	Age (years)						***P*** value
	< 40 ***N*** = 5	40–49 ***N*** = 36	50–59 ***N*** = 86	60–69 N = 86	70–79 ***N*** = 68	> 79 ***N*** = 9	
Basal serum cortisol (nmol/l)	a	a	371 (312–439)	375 (323–458)	403 (324.5–457.25)	392 (317–430)	0.489
Serum cortisol after ODST (nmol/l)^a^	28 (27.6–28)	30.3 (27.6–38)	35.35 (30.9–39.28)	34.05 (28.78–43.28)	36.85 (29.28–41.33)	30.3 (27.6–38.95)	0.183
Basal DHEAS (μmol/L)	a	2.15 (1.7–2.8)	1.7 (1.2–3.2)	1.55 (0.83–2.1)	1.3 (0.81–2.1)	1.15 (0.53–2.45)	**0.002**
DHEAS after ODST (μmol/L)	a	0.65 (0.4–1.2)	0.9 (0.4–1.8)	0.69 (0.3–1.2)	0.6 (0.34–0.93)	1.15 (0.23–1.95)	**0.041**
Aldosteron (nmol/l)	a	0.18 (0.15–0.2)	0.14 (0.1–0.23)	0.17 (0.11–0.26)	0.2 (0.11–0.26)	0.1 (0.06–0.26)	0.278
Plasma Renin Activity – PRA (μg/l/h)	a	0.41 (0.23–0.89)	0.39 (0.15–1.28)	0.27 (0.11–1.5)	0.54 (0.26–1.87)	0.31 (0.01–0.31)	0.421
TSH (mE/l)	1.35 (1.27–1.35)	1.39 (1.19–3.04)	2.11 (1.24–3.26)	6.5 (5.6–7.63)	1.33 (0.83–4.92)	0.46 (0.21–0.79)	**< 0.001** Pairwise comparisons 50–59 vs. > 79 *P* = 0.005 60–69 vs. > 79 *P* < 0.001 60–69 vs. 70–79 *P* = 0.003 40–49 vs. 60–69 *P* = 0.007 50–59 vs. 60–69 *P* = 0.024
Body mass (kg)	92.45 (54.75–143.95)	75.3 (65.9–86)	83.5 (72.3–93.85)	78.5 (73.5–90)	80.45 (65.7–88.9)	71 (63.5–82)	**0.031**
BMI (kg/m^2^)	35.45 (20.46–52.53)	28.57 (24.13–31.2)	29.04 (25.77–32.54)	28.56 (25.8–33.35)	28.2 (24.98–31.23)	28.04 (25.59–30.53)	0.715
Systolic blood pressure (mm Hg)	120 (118.5–160)	127.5 (120–140)	135 (120–145)	141.5 (126.5–151.5)	145 (126–155)	155 (152–170)	**< 0.001** Pairwise comparisons 40–49 vs. > 79 *P* = 0.003 50–59 vs. > 79 *P* = 0.007
Diastolic blood pressure (mm Hg)	85 (80–97.5)	77 (73–86.25)	78 (70–87)	75 (70–83.5)	74 (65–80)	88 (80–98)	**0.002** Pairwise comparisons 70–79 vs. > 79 *P* = 0.014
Heart rate	94 (69.5–100.5)	80 (71.5–90)	73.5 (68–90.25)	72.5 (63.25–81.75)	75 (67–87.75)	70 (66–87)	0.258
Fasting glucose (mmol/liter)	4.5 (4.4–5.2)	4.9 (4.6–5.5)	5.3 (5–6.2)	5.55 (5.03–6.2)	5.4 (5–6.2)	5.6 (5.05–6.35)	**0.003** Pairwise comparisons 40–49 vs. 60–69 *P* = 0.015
Total cholesterol (mmol/liter)	4.5 (3.93–5.83)	5.3 (4.88–5.83)	5.55 (4.5–6.4)	4.95 (4.33–5.5)	4.7 (4.1–5.7)	4.45 (3.58–5.58)	**0.038**
HDL (mmol/liter)	1.3 (0.95–1.95)	1.2 (1–1.43)	1.3 (1.1–1.5)	1.3 (1.1–1.58)	1.2 (1.1–1.6)	1.1 (0.95–1.15)	0.419
LDL (mmol/liter)	2.35 (1.9–3.55)	3.4 (3–3.9)	3.55 (2.7–4.3)	2.9 (2.33–3.58)	2.8 (2.2–3.5)	2.75 (1.95–3.65)	**0.002** Pairwise comparisons 50–59 vs. 70–79 *P* = 0.008 50–59 vs. 60–69 *P* = 0.047
Triglycerides (mmol/liter)	1.3 (0.85–4.45)	1.6 (1.08–2.03)	1.7 (1.3–2.2)	1.5 (1.2–2.2)	1.3 (1.05–2.05)	1.55 (1.08–2.08)	0.417
Creatinine (mmol/liter)	68.5 (61.5–75.5)	62 (57–71.5)	71 (64–79)	69 (61–84)	77 (64–95)	72 (64.5–86.5)	**0.006** Pairwise comparisons 40–49 vs. 70–79 *P* = 0.001
Sodium (mmol/liter)	142.5 (139.75–143)	141 (140–142.5)	142 (141–143)	142 (141–144)	142 (140.75–143)	143 (142.5–144)	0.058

for 70 patients, data were provided as below 27.6, for 20 below 28 and for 1 below 31.3

^a^data available for only 1 patient or no patients

Data are given as Median (25–75%)

### ACS group stratified by age

In ACS group, younger subjects significantly differ from older in blood pressure, cholesterol, LDL, fasting glucose, creatinine. The data are provided in Table 3. TSH levels differed significantly among age groups (*P* < 0.001), with highest values in patients aged 60–69 years and lowest values in patients aged 70–79 years. There were no differences in leukocyte concentration (*P* = 0.425).

**Table 3:** Stratification of hormonal, antropometric, clinical, radiologic, biochemical and metabolic parameters in ACS patients by age

Variable	Age (years)						***P*** value
	< 40 N = 3	40–49 ***N*** = 11	50–59 ***N*** = 40	60–69 ***N*** = 37	70–79 ***N*** = 36	> 79 ***N*** = 15	
Basal serum cortisol (nmol/l)	^b^	339 (239-)	372 (320.5–565.5)	444 (362.5–510.5)	478.5 (372–606.5)	475 (451–604)	0.123
Serum cortisol after ODST (nmol/l)^a^	56 (54.1-)	62.3 (52–92.7)	70.25 (57.45–93.18)	67.9 (54.95–94.35)	62.95 (56.8–84.28)	67 (52.4–76.4)	0.774
Basal DHEAS (μmol/L)	^b^	1.85 (0.5–3)	0.9 (0.43–1.8)	0.9 (0.5–1)	0.44 (0.38–1.03)	0.9 (0.4–1.68)	0.445
DHEAS after ODST (μmol/L)	^b^	0.8 (0.2–1.2)	0.5 (0.3–1.1)	0.5 (0.3–0.85)	0.5 (0.2–0.7)	1 (0.25–1.8)	0.716
Aldosteron (nmol/l)	^b^	0.31 (0.13–0.62)	0.2 (0.11–0.28)	0.21 (0.14–0.42)	0.21 (0.07–0.35)	0.23 (0.14–0.27)	0.870
Plasma Renin Activity – PRA (μg/l/h)	^b^	0.21 (0.06–2.69)	0.49 (0.15–1.06)	0.68 (0.34–2.98)	0.94 (0.34–2.32)	0.61 (0.18–1.97)	0.719
TSH (mE/l)	4.51 (2.4-)	0.39 (0.34–1.31)	1.37 (0.71–2.36)	6.15 (5.73–7.48)	0.8 (0.48–5.85)	1.26 (0.51–3.15)	**0.001** Pairwise comparisons 40–49 vs. 60–69 *P* = 0.002 50–59 vs. 60–69 *P* = 0.021 60–69 vs. 70–79 *P* = 0.025
Body mass (kg)	75 (74.6-)	67.3 (62.3–75)	83.1 (67.5–92.2)	73 (62.3–89.3)	76 (67.7–80.8)	70.15 (64.48–82.85)	0.061
BMI (kg/m^2^)	27.76 (25.81-)	25.27 (22.58–26.91)	28.91 (25.61–31.9)	29.34 (22.98–32.12)	27.59 (26.13–33.46)	28.55 (25.61–30.92)	0.227
Systolic blood pressure (mm Hg)	122 (119–122)	120 (115–140)	140.5 (125.75–150.75)	145 (112–150)	146.5 (131.25–164.5)	150 (135–160)	**0.038**
Diastolic blood pressure (mm Hg)	82 (76–82)	75 (75–90)	80 (75–90)	73 (68–85)	75 (70–80)	74 (65–82)	**0.037**
Heart rate	91 (51–91)	80.5 (70.25–94.5)	78 (65–84.75)	77 (67–84)	70 (66.25–84.5)	82 (71.75–90.5)	0.637
Fasting glucose (mmol/liter)	4.7 (4.6-)	4.6 (4.28–5.1)	5.15 (4.93–5.98)	5.5 (5.1–6.38)	5.4 (5–5.98)	6.15 (5.33–6.55)	**0.003** Pairwise comparisons 40–49 vs. 60–69 *P* = 0.021 40–49 vs. > 79 *P* = 0.006
Total cholesterol (mmol/liter)	5.4 (5.3-)	5.05 (5-)	5.7 (4.7–6.5)	5 (4.1–5.9)	4.6 (4–5.2)	5 (3.6–5.3)	**0.019** Pairwise comparisons 50–59 vs. 70–79 *P* = 0.014
HDL (mmol/liter)	1.3 (1-)	1.45 (1.3-)	1.2 (1–1.6)	1.3 (1.1–1.9)	1.2 (1.1–1.5)	1.05 (0.75–1.1)	0.125
LDL (mmol/liter)	3.6 (3.2-)	2.85 (2.7-)	3.6 (2.9–4.4)	3 (2.5–3.5)	2.7 (2.1–3)	2.7 (1.4–3.7)	**0.021** Pairwise comparisons 50–59 vs. 70–79 *P* = 0.010
Triglycerides (mmol/liter)	2.7 (1.2-)	1.55 (1.1-)	1.6 (1–2)	1.6 (1.2–2)	1.7 (1.2–2.1)	1.9 (1.15–2.85)	0.660
Creatinine (mmol/liter)	69 (64-)	69 (59–74)	67.5 (60.5–74.75)	80 (71–90)	74 (67–92)	79.5 (68.5–96)	**0.006** Pairwise comparisons 50–59 vs. 60–69 *P* = 0.023
Sodium (mmol/liter)	141 (137-)	141 (139–143)	142 (140–144)	142 (141–143)	142 (141–144)	141.5 (139.75–144)	0.630

^a^for 70 patients, data were provided as below 27.6, for 20 below 28 and for 1 below 31.3

^b^data available for only 1 patient or no patients

Data are given as Median (25–75%)

## Stratification of patients with NFAI and ACS by BMI

There was no significant difference between NFAI and ACS groups regarding BMI (*P* = 0.287). BMI was not correlated with serum cortisol after ODST (Spearman’s rho = − 0.041, *P* = 0.436) in the whole study population.

### NFAI group stratified by BMI

When stratified by BMI (≤ 25 kg/m^2^, 25–30 kg/m^2^ and > 30 kg/m^2^), patients with NFAI and higher BMI, had higher fasting glucose (*P* < 0.001, pairwise comparison BMI ≤ 25 kg/m^2^ vs. BMI > 30 kg/m^2^*P* < 0.001, 25–30 kg/m^2^*P* = 0.050), lower HDL (*P* = 0.009, pairwise comparison BMI ≤ 25 kg/m^2^ vs. BMI > 30 kg/m^2^*P* = 0.007), higher triglycerides (*P* = 0.001, pairwise comparison BMI ≤ 25 kg/m^2^ vs. BMI > 30 kg/m^2^*P* < 0.001), higher creatinine (*P* = 0.008, pairwise comparison BMI ≤ 25 kg/m^2^ vs. BMI > 30 kg/m^2^*P* = 0.032, 25–30 kg/m^2^*P* = 0.050) (*P* = 0.023) and higher leukocytes (*P* = 0.014, pairwise comparison BMI ≤ 25 kg/m^2^ vs. BMI > 30 kg/m^2^*P* = 0.019). There were significantly more patients with diabetes mellitus in higher BMI groups (*P* = 0.002).

### ACS group stratified by BMI

When stratified by BMI patients with ACS and different BMI (≤ 25 kg/m^2^ vs. 25–30 kg/m^2^ vs. > 30 kg/m^2^), differed in TSH (*P* = 0.006, pairwise comparison BMI 25–30 kg/m^2^ vs. BMI > 30 kg/m^2^*P* = 0.005), HDL (*P* = 0.006, pairwise comparison BMI 25–30 kg/m^2^ vs. BMI > 30 kg/m^2^*P* = 0.005) and creatinine (*P* = 0.012, pairwise comparison BMI ≤ 25 kg/m^2^ vs. BMI > 30 kg/m^2^*P* = 0.0471, 25–30 kg/m^2^ vs. BMI > 30 kg/m^2^*P* = 0.011). Patients with ACS across the three BMI groups (≤ 25 kg/m^2^, 25–30 kg/m^2^ and > 30 kg/m^2^) did not differ in age, basal cortisol, basal DHEAS and DHEAS after ODST, aldosterone, PRA, TSH, blood pressure, heart rate, fasting glucose, cholesterol, triglycerides, LDL, sodium and leukocytes. There was no significant difference in gender distribution between groups (*P* > 0.05). Regression analysis did not show any significant correlation between BMI and presence of diabetes mellitus in this group (OR = 1.00, 95% CI = 0.90–1.12, *P* = 0.969).

## Prevalence of NFAI and ACS patients stratified by age and BMI

**Fig. 2:**
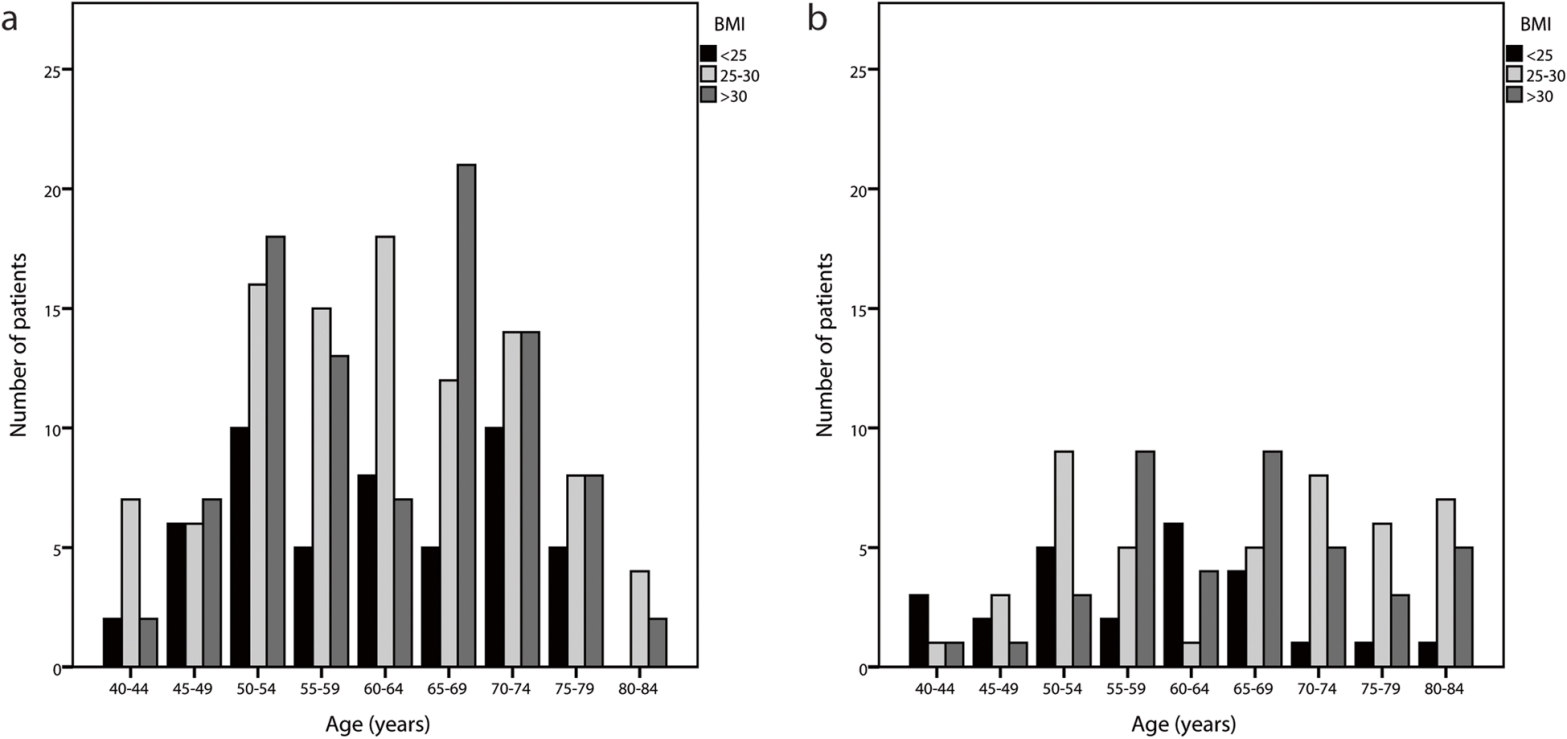
Prevalence of AI in NFAI and ACS patients stratified by age and BMI *. a: NFAI patients (*N* = 248, 42 missing BMIs), b: ACS patients (*N* = 114, 28 missing BMIs). *The data analyses for patients below 40 years and above 84 years are truncated because less than 5 subjects were included within those age-subgroups

We observed 68–87% higher prevalence of AI in all age groups in both NFAI and ACS in obese/overweight compared to normal weight subjects (Fig. 2).

### NFAI group stratified by age and BMI

BMI > 25 kg/m^2^ had 100% of patients aged 25–29 years, 81.8% patients aged 40–44 years, 68.4% patients aged 45–49 years, 77.3% patients aged 50–54 years, 81.8% patients aged 55–59 years, 75.8% patients aged 60–64 years, 86.8% patients aged 65–69 years, 73.7% patients aged 70–74 years, 76.2% patients aged 75–79 years, 68.4% patients aged 80–84 years. Only in 2 age groups at the tails of age distribution, AIs were more common in normal weight than in overweight/obese group (2/2 patients in age group 30–34 years 66.7% had BMI ≤ 25 kg/m^2^ and 1/1 patient aged over 84 years had BMI ≤ 25 kg/m^2^. However, number of the subjects in these two groups were neglectable (Fig. 2).

### ACS group stratified by age and BMI

In group with ACS, BMI > 25 kg/m^2^ had 100% of patients aged 25–29 and 35–39 years, 40% of patients aged 40–44 years, 66.7% % of patients aged 45–49 years, 70.6% of patients aged 50–54 years, 87.5% of patients aged 55–59 years, 45.5% of patients aged 60–64 years, 77.8% of patients aged 65–69 years, 92.9% of patients aged 70–74 years, 90% of patients aged 75–79 years, 92.3% of patients aged 80–84 years. The only patient aged over 84 years had BMI ≤ 25 kg/m^2^ (Fig. 2).

## Discussion

We observed 68–87% higher prevalence of NFAI and ACS across the age distribution in overweight and obese subject when compared to normal weight subjects. Patients with ACS were older, with higher basal cortisol and aldosterone and lower basal and suppression DHEAS. Adrenal incidentalomas with ACS were significantly larger than NFAI. Interestingly, ACS group had lower mean body mass than NFAI and did not differ in BMI, blood pressure, heart rate, lipid profile, fasting glucose and presence of diabetes mellitus type 2 when compared with NFAI. By contrast, some metabolic syndrome-related traits were associated with higher BMI and age, in particular in NFAI group.

So far, the relationship between obesity, metabolic traits and AIs in clinical studies have been discussed mainly in one direction: as the consequences of mild autonomous cortisol secretion in ACS [7, 20–22] and as possible consequences of minimal hormonal secretion not detectable by current diagnostic methods in NFAI [23–25]. However, the observed anabolic and mitogenic effects of insulin on adrenal cortex from preclinical models led to the hypothesis of the potential existence of bilateral relationship between obesity and AIs [7].

In clinical studies, obesity as a primary cause of AI and as a main risk estimator of metabolic burden in patients with AI remained largely unaddressed. Our study population demonstrated that significantly more patients with AIs were overweight/obese than normal weight across the age distribution. These were observed in both, ACS group, where obesity has been traditionally linked to autonomous cortisol secretion [7, 21, 22], as well as in NFAI group, where this link has not been as well supported. In line with other studies [26, 27], there was female predominance in gender distribution across all ages, which was, possibly due to more imaging in women than men. In addition, this observation could also coincide with obesity being more prevalent in women than in men in general population [27–29].

As opposed to the traditional predisposition that autonomous cortisol secretion in ACS led to obesity and adverse metabolic profile, our ACS group had lower mean body mass than NFAI and did not differ in BMI, blood pressure, heart rate, lipid profile, fasting glucose and presence of diabetes mellitus type 2 when compared with NFAI. Similarly, recently published study of Spanish retrospective cohort including 149 patients with AI reported that the prevalence of obesity was lower in ACS than in NFAI [26] and that the differences in high blood pressure, lipids, cardiovascular and cerebrovascular disease did not reached statistical significance if they used the ODMT-cut off of 1.8 μg/dl to differentiated between ACS and NFAI [26]. Based on that cut off, the difference had only reached statistical significance for the presences of diabetes that was more frequent in ACS [26]. Taken together, the differentiation based on the low-grade incomplete post-dexamethasone cortisol suppression does not seem to be a reliable predictor of metabolic alterations in AIs. It might be useful in combination with other clinical and/or biochemical criteria including elevated urinary free cortisol and reduced ACTH, but so far there is no consensus on the the potential scoring index [30, 31].

By contrast to the similarity of metabolic profiles in ACS and NFAI, some metabolic syndrome-related traits in our study population were rather associated with higher BMI and older age in both groups, particularly in NFAI. Patients with NFAI and higher BMI, had higher fasting glucose, lower HDL, higher triglycerides, higher leukocytes and higher presence of diabetes. Younger patients with NFAI had lower blood pressure, lower body mass, LDL and fasting glucose. In ACS group, younger subjects had significantly lower systolic blood pressure and fasting glucose, whereas there were no benefits of younger age in lipid profile. Further longitudinal studies should consider the roles of age and BMI as potential risk estimators of cardiometabolic and cerebrovascular burden in patients with ACS and NFAI.

Since unilateral and bilateral AIs might have distinct pathophysiologies [32], we also compared characteristics of unilateral and bilateral AIs in both groups. In NFAI group less than 20% of AIs were bilateral. One-sided NFAI did not differ from bilateral NFAI in any of patients’ or tumors’ characteristics. In ACS almost 40% of AIs were bilateral. Patients with one-sided ACS had lower cholesterol and smaller left-sided unilateral AIs when compared to left sided AIs in patients with bilateral ACS. One-sided ACS did not differ from bilateral ACS in any other anthropometric, clinical, hormonal, biochemical or radiologic characteristics. The higher frequency of bilateral tumor in ACS compared to NFAI is in agreement with meta-analysis [32] confirming that bilateral AIs are most frequently correlated with cortisol production [33]. Furthermore, unilateral vs. bilateral ratio did not change across BMI distribution in either of our groups. Given that IR and related effects of chronic insulin hypersecretion are expected to have a systemic effect, it is of interest that most AIs across BMI distributions are unilateral, as opposed to bilateral. It was previously hypothesized that the primary cause of the focal cell hyperplasia, characteristic for adrenal nodular unilateral disease, is related to the intrinsic heterogeneity of target cells in responding to anabolic insulin and other growth stimulating factors [34].

As expected, basal cortisol was higher in ACS than in NFAI. In addition, patients with ACS in our study population had significantly higher basal aldosterone than those in NFAI. Recent analysis using steroid metabolomics demonstrated that glucocorticoid excess is a highly prevalent feature in primary aldosteronism implying that the traditional division into cortisol and mineralocorticoid producing adrenal tumors is not as clear cut as previously assumed [35]. Further studies with highly sensitive diagnostic and biomarker tools will have to assess the clinical impact of the potential “Connshing” syndrome with co-secretion of gluco- and mineralo-corticoids. Furthermore, in line with other studies [36] our study population with ACS had significantly lower basal DHEAS levels. In autonomous adrenal cortisol excess, DHEAS is usually downregulated due to reduced hypothalamic-pituitary feedback to the adrenal glands in response to the glucocorticoid excess [35]. Indeed, the potential utility of DHEAS to reflect ambient ACTH levels over a longer time interval has led to DHEAS being proposed as an indicator of ACS [36].

Some limitations of our study should be considered. The results are limited due to retrospective analysis of patients’ records and some incomplete data. The comparison was made only for NFAI and ACS based on one cut off considered as low-grade incomplete post-dexamethasone cortisol suppression (cortisol levels post dexamethasone > 50 nmol/L). Further subdivision including second cut off for cortisol levels post dexamethasone > 138 nmol/L was not performed since only 14 out of 142 patients in ACS would have been characterized for this subgroup. Moreover, there might be a selection bias since the obesity and its complications, in particular among younger patients could be the reason for performing a CT scan and the detection of AIs. However, we excluded all symptomatic patients including some of those imaging indications that are closely related to obesity such as symptomatic cholecystolithiasis and pancreatitis.

## Conclusions

By our knowledge, comparisons of the patients’ and tumor’s characteristics according to the triple stratification based on incomplete post-dexamethasone cortisol suppression, age and BMI have not yet been performed. Although our study does not prove causality of relationship between obesity and development of AIs, it eloquently portrays the importance of considering the primary role of BMI when addressing the prevalence as well as patients’ and tumor’s characteristics in both, ACS and NFAI groups. We encourage longitudinal studies aiming to investigate the potential development of AIs and potential growth of pre-existent AIs in subjects with obesity. Furthermore, interventional studies in overweight/obese patients with AI should assess the potential impact of insulin sensitizers and weight reduction on the size, growth, functionality and metabolic consequences of AIs. Translational studies evaluating the molecular phenotype of AI related to relevant signaling cascades in IGF-1 and MAPK pathways might also provide further insights into this potential bidirectional relationship between obesity and AIs.

## Data Availability

The datasets used and/or analyzed during the current study are available from the corresponding author on reasonable request.

## Abbreviations

ACS: Autonomous cortisol secreting adrenal incidentaloma
ACTH: Adrenocorticotropic hormone
AI: Adrenal incidentaloma
BMI: Body mass indexe
CT: Computed tomography
DHEAS: Dehydroepiandrosterone sulfate
HDL: High density lipoprotein
HGHR: Hepatic growth hormone receptor
HU: Hounsfield unit
IGF 1: Insulin like growth factor 1
IR: Insulin resistence
LDL: Low density lipoprotein
MAPK: Mitogen-activated protein kinase
MRI: Magnetic resonance imaging
N: Numerus
NFAI: Nonfunctional adrenal incidentaloma
ODST: Cortisol after overnight dexamethasone suppression test
P: *P*-value
PRA: Plasma renin activity
TSH: Thyroid stimulating hormone
